# Bis(bicyclo­[2.2.1]hept-5-ene-2-carboxyl­ato)-1κ^2^ *O*,*O*′;4κ^2^ *O*,*O*′-di-μ_2_-chlorido-1:2κ^2^ *Cl*;3:4κ^2^ *Cl*-octa­methyl-1κ^2^ *C*,2κ^2^ *C*,3κ^2^ *C*,4κ^2^ *C*-di-μ_3_-oxido-1:2:3κ^3^ *O*;2:3:4κ^3^ *O*-tetra­tin(IV)

**DOI:** 10.1107/S1600536811023452

**Published:** 2011-06-25

**Authors:** Yupo Ren

**Affiliations:** a Department of Pathology, Liaocheng People’s Hospital, Liaocheng, Shandong 252000, People’s Republic of China

## Abstract

In the title compound, [Sn_4_(CH_3_)_8_(C_8_H_9_O_2_)_2_Cl_2_O_2_], the tetra­nuclear complex mol­ecule has crystallographically imposed inversion symmetry. The coordination polyhedron about the two central Sn atoms is distorted trigonal–bipyramidal, whilst the two peripheral metal atoms bonded to the carboxyl­ate groups have a distorted octa­hedral coordination geometry. In the crystal, mol­ecules are connected by long Sn⋯O contacts [3.139 (11) Å], forming chains along [011].

## Related literature

For the biological activity of organotin compounds, see: Dubey & Roy (2003 ▶). For a related structure, see: Li *et al.* (2006 ▶). 
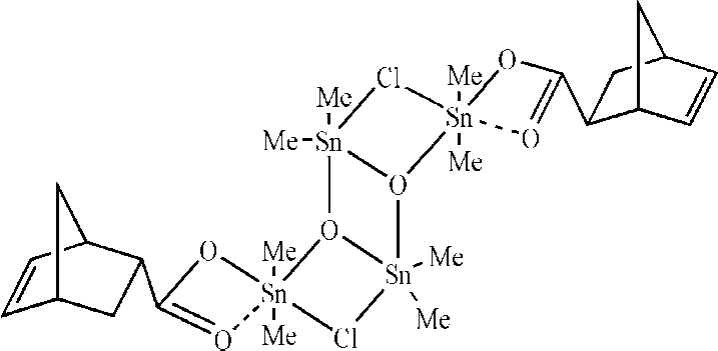


## Experimental

### 

#### Crystal data

[Sn_4_(CH_3_)_8_(C_8_H_9_O_2_)_2_Cl_2_O_2_]
*M* *_r_* = 972.24
Triclinic, 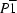

*a* = 9.3685 (12) Å
*b* = 9.8651 (13) Å
*c* = 9.9103 (15) Å
α = 109.779 (2)°
β = 96.340 (1)°
γ = 97.204 (1)°
*V* = 843.5 (2) Å^3^
*Z* = 1
Mo *K*α radiation
μ = 3.12 mm^−1^
*T* = 298 K
0.13 × 0.11 × 0.05 mm

#### Data collection

Siemens SMART CCD area-detector diffractometer
Absorption correction: multi-scan (*SADABS*; Sheldrick, 1996 ▶) *T*_min_ = 0.687, *T*_max_ = 0.860
4366 measured reflections
2915 independent reflections
1818 reflections with *I* > 2σ(*I*)
*R*_int_ = 0.024

#### Refinement

*R*[*F* ^2^ > 2σ(*F* ^2^)] = 0.047
*wR*(*F* ^2^) = 0.130
*S* = 1.02
2915 reflections
163 parameters
H-atom parameters constrained
Δρ_max_ = 0.86 e Å^−3^
Δρ_min_ = −0.64 e Å^−3^

### 

Data collection: *SMART* (Siemens, 1996 ▶); cell refinement: *SAINT* (Siemens, 1996 ▶); data reduction: *SAINT*; program(s) used to solve structure: *SHELXS97* (Sheldrick, 2008 ▶); program(s) used to refine structure: *SHELXL97* (Sheldrick, 2008 ▶); molecular graphics: *SHELXTL* (Sheldrick, 2008 ▶); software used to prepare material for publication: *SHELXTL*.

## Supplementary Material

Crystal structure: contains datablock(s) I, global. DOI: 10.1107/S1600536811023452/rz2611sup1.cif

Structure factors: contains datablock(s) I. DOI: 10.1107/S1600536811023452/rz2611Isup2.hkl

Additional supplementary materials:  crystallographic information; 3D view; checkCIF report

## Figures and Tables

**Fig. 1.**
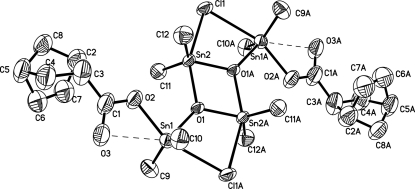
The molecular structure of the title compound, showing 50% probability displacement ellipsoids. H atoms have been omitted for clarity. Symmetry code: (A) 1-x, 1-y, 2-z.

**Fig. 2.**
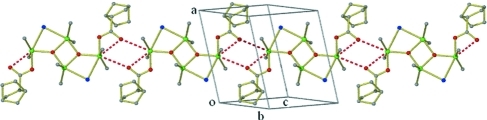
View of the one-dimensional chain structure in the title compound.

## supplementary crystallographic information

### Comment

In recent years, organotin compounds have attracted more and more attention due to their wide range of industrial applications and biological activities (Dubey & Roy, 2003). As a part of our ongoing investigations in this field, we have synthesized the title compound and present its crystal structure here.

The tetranuclear complex molecule of the title compound (Fig. 1) has crystallographically imposed inversion symmetry. The tin atoms have two different coordination modes, one atom (Sn2) is coordinated in a distorted trigonal-bipyramidal geometry by one µ_3_oxo oxygen atom and two methyl groups forming the equatorial plane, and by an µ_3_oxo oxygen atom and the chloride anion at the apices; the other tin metal (Sn1) has a distorted octahedral coordination geometry, with three O atoms and one Cl atom in equatorial positions and the axial position occupied by two methyl groups. The Sn—O bond distances involving the carboxylate group (Sn1—O2 = 2.115 (8) Å; Sn1—O3 = 2.699 (9) Å) are comparable to those found in a related organotin carboxylate (Li *et al.*, 2006). The shortest Sn···Sn separation within the Sn_4_ core is 3.2898 (10) Å. In the crystal structure, complex molecules are connected by long Sn···O interaction (Sn1···O3^i^ = 3.139 (11) Å; symmetry code: (i) 1-x, -y, 1-z) into one-dimensional chains parallel to the [011] direction (Fig. 2).

### Experimental

The reaction was carried out under a nitrogen atmosphere. Bicyclo[2.2.1]heptane-2-carboxylic acid (1 mmol) and sodium ethoxide (1 mmol) were added to a stirred solution of benzene (30 ml) in a Schlenk flask After stirring the solution for 30 min, dimethyltin dichloride (2 mmol) was added and the reaction mixture was stirred for 12 h at room temperature. The resulting clear solution was evaporated under vacuum. The product was crystallized from a solution of diethyl ether to yield colourless crystals of the title compound suitable for X-ray analysis (yield: 76%). Anal. Calcd (%) for C_24_H_42_Cl_2_O_6_Sn_4_ (Mr = 972.33): C, 29.65; H, 4.35. Found (%): C, 29.47; H, 4.52.

### Refinement

The H atoms were positioned geometrically, with C—H = 0.93–0.98 Å, and refined as riding on their parent atoms, with *U*_iso_(H) = 1.2 *U*_eq_(C) or 1.5 *U*_eq_(C) for the methyl groups.

### Crystal data

**Table:**

[Sn_4_(CH_3_)_8_(C_8_H_9_O_2_)_2_Cl_2_O_2_]	*Z* = 1
*M**_r_* = 972.24	*F*(000) = 468
Triclinic, *P*1	*D*_x_ = 1.914 Mg m^−^^3^
Hall symbol: -P 1	Mo *K*α radiation, λ = 0.71073 Å
*a* = 9.3685 (12) Å	Cell parameters from 1509 reflections
*b* = 9.8651 (13) Å	θ = 2.2–25.2°
*c* = 9.9103 (15) Å	µ = 3.12 mm^−^^1^
α = 109.779 (2)°	*T* = 298 K
β = 96.340 (1)°	Block, colourless
γ = 97.204 (1)°	0.13 × 0.11 × 0.05 mm
*V* = 843.5 (2) Å^3^	

### Data collection

**Table:**

Siemens SMART CCD area-detector diffractometer	2915 independent reflections
Radiation source: fine-focus sealed tube	1818 reflections with *I* > 2σ(*I*)
graphite	*R*_int_ = 0.024
φ and ω scans	θ_max_ = 25.0°, θ_min_ = 2.2°
Absorption correction: multi-scan (*SADABS*; Sheldrick, 1996)	*h* = −6→11
*T*_min_ = 0.687, *T*_max_ = 0.860	*k* = −11→10
4366 measured reflections	*l* = −11→11

### Refinement

**Table:**

Refinement on *F*^2^	Primary atom site location: structure-invariant direct methods
Least-squares matrix: full	Secondary atom site location: difference Fourier map
*R*[*F*^2^ > 2σ(*F*^2^)] = 0.047	Hydrogen site location: inferred from neighbouring sites
*wR*(*F*^2^) = 0.130	H-atom parameters constrained
*S* = 1.02	*w* = 1/[σ^2^(*F*_o_^2^) + (0.0634*P*)^2^] where *P* = (*F*_o_^2^ + 2*F*_c_^2^)/3
2915 reflections	(Δ/σ)_max_ = 0.001
163 parameters	Δρ_max_ = 0.86 e Å^−^^3^
0 restraints	Δρ_min_ = −0.64 e Å^−^^3^

### Special details

**Table:**

Geometry. All e.s.d.'s (except the e.s.d. in the dihedral angle between two l.s. planes) are estimated using the full covariance matrix. The cell e.s.d.'s are taken into account individually in the estimation of e.s.d.'s in distances, angles and torsion angles; correlations between e.s.d.'s in cell parameters are only used when they are defined by crystal symmetry. An approximate (isotropic) treatment of cell e.s.d.'s is used for estimating e.s.d.'s involving l.s. planes.
Refinement. Refinement of *F*^2^ against ALL reflections. The weighted *R*-factor *wR* and goodness of fit *S* are based on *F*^2^, conventional *R*-factors *R* are based on *F*, with *F* set to zero for negative *F*^2^. The threshold expression of *F*^2^ > σ(*F*^2^) is used only for calculating *R*-factors(gt) *etc*. and is not relevant to the choice of reflections for refinement. *R*-factors based on *F*^2^ are statistically about twice as large as those based on *F*, and *R*- factors based on ALL data will be even larger.

### Fractional atomic coordinates and isotropic or equivalent isotropic displacement parameters (Å^2^)

**Table:**

	*x*	*y*	*z*	*U*_iso_*/*U*_eq_	
Sn1	0.52583 (8)	0.20738 (8)	0.72575 (8)	0.0642 (3)	
Sn2	0.34860 (7)	0.52430 (7)	0.91708 (7)	0.0541 (2)	
Cl1	0.2281 (3)	0.7487 (4)	1.0584 (3)	0.0893 (9)	
O1	0.5103 (6)	0.3891 (6)	0.8952 (6)	0.0567 (16)	
O2	0.3315 (9)	0.2563 (10)	0.6371 (9)	0.104 (3)	
O3	0.3517 (12)	0.0621 (12)	0.4673 (11)	0.123 (3)	
C1	0.2708 (19)	0.160 (2)	0.5170 (19)	0.126 (6)	
C2	0.114 (2)	0.312 (2)	0.436 (2)	0.147 (7)	
H2	0.1241	0.4026	0.5205	0.177*	
C3	0.1132 (19)	0.168 (2)	0.4573 (18)	0.137 (6)	
H3	0.0508	0.1632	0.5294	0.164*	
C4	0.042 (2)	0.057 (2)	0.3123 (19)	0.152 (7)	
H4A	0.1041	−0.0144	0.2773	0.183*	
H4B	−0.0502	0.0065	0.3211	0.183*	
C5	0.017 (2)	0.135 (2)	0.2080 (19)	0.139 (6)	
H5	−0.0452	0.0854	0.1128	0.166*	
C6	0.183 (2)	0.175 (2)	0.223 (2)	0.156 (7)	
H6	0.2425	0.1242	0.1621	0.187*	
C7	0.226 (2)	0.295 (2)	0.337 (2)	0.153 (7)	
H7	0.3122	0.3604	0.3543	0.183*	
C8	−0.0092 (18)	0.277 (2)	0.3130 (18)	0.140 (6)	
H8A	−0.0047	0.3524	0.2702	0.168*	
H8B	−0.1029	0.2657	0.3446	0.168*	
C9	0.6728 (14)	0.2796 (14)	0.6114 (13)	0.101 (4)	
H9A	0.7045	0.3833	0.6575	0.152*	
H9B	0.6262	0.2576	0.5134	0.152*	
H9C	0.7556	0.2311	0.6106	0.152*	
C10	0.4421 (14)	0.0339 (13)	0.7839 (13)	0.095 (4)	
H10A	0.4365	0.0689	0.8857	0.142*	
H10B	0.5048	−0.0379	0.7639	0.142*	
H10C	0.3464	−0.0094	0.7289	0.142*	
C11	0.4013 (13)	0.6224 (13)	0.7674 (12)	0.086 (3)	
H11A	0.3294	0.6805	0.7552	0.129*	
H11B	0.4031	0.5479	0.6756	0.129*	
H11C	0.4954	0.6837	0.8028	0.129*	
C12	0.1694 (12)	0.3767 (14)	0.9197 (13)	0.098 (4)	
H12A	0.1857	0.2786	0.8743	0.147*	
H12B	0.0834	0.3910	0.8676	0.147*	
H12C	0.1567	0.3928	1.0184	0.147*	

### Atomic displacement parameters (Å^2^)

**Table:**

	*U*^11^	*U*^22^	*U*^33^	*U*^12^	*U*^13^	*U*^23^
Sn1	0.0789 (5)	0.0509 (5)	0.0573 (5)	0.0212 (4)	0.0060 (4)	0.0105 (4)
Sn2	0.0483 (4)	0.0559 (5)	0.0558 (4)	0.0153 (3)	−0.0009 (3)	0.0177 (3)
Cl1	0.0721 (18)	0.088 (2)	0.092 (2)	0.0425 (15)	−0.0049 (15)	0.0080 (17)
O1	0.057 (4)	0.052 (4)	0.056 (4)	0.028 (3)	−0.001 (3)	0.009 (3)
O2	0.113 (7)	0.093 (7)	0.090 (6)	0.043 (5)	−0.023 (5)	0.013 (5)
O3	0.142 (9)	0.113 (9)	0.091 (7)	0.039 (7)	−0.028 (6)	0.016 (6)
C1	0.132 (14)	0.121 (14)	0.103 (12)	0.041 (11)	−0.031 (10)	0.023 (11)
C2	0.154 (17)	0.134 (17)	0.121 (15)	0.047 (13)	−0.017 (13)	0.009 (12)
C3	0.163 (16)	0.124 (15)	0.108 (13)	0.021 (12)	−0.021 (11)	0.036 (12)
C4	0.157 (16)	0.137 (16)	0.131 (15)	0.024 (12)	−0.027 (12)	0.022 (14)
C5	0.146 (16)	0.137 (16)	0.108 (13)	0.033 (12)	−0.038 (11)	0.028 (12)
C6	0.147 (17)	0.154 (18)	0.122 (15)	0.036 (13)	−0.005 (12)	−0.002 (13)
C7	0.150 (17)	0.141 (18)	0.132 (16)	0.020 (13)	−0.001 (14)	0.012 (14)
C8	0.141 (14)	0.138 (16)	0.124 (14)	0.065 (11)	−0.025 (11)	0.025 (12)
C9	0.125 (11)	0.091 (10)	0.084 (9)	0.025 (8)	0.029 (8)	0.019 (8)
C10	0.109 (10)	0.075 (9)	0.093 (9)	0.009 (7)	0.003 (7)	0.028 (7)
C11	0.105 (9)	0.084 (9)	0.086 (8)	0.034 (7)	0.019 (7)	0.044 (7)
C12	0.080 (8)	0.089 (9)	0.111 (10)	−0.002 (7)	0.009 (7)	0.026 (8)

### Geometric parameters (Å, °)

**Table:**

Sn1—O1	2.034 (6)	C4—C5	1.49 (2)
Sn1—C10	2.075 (11)	C4—H4A	0.9700
Sn1—C9	2.082 (11)	C4—H4B	0.9700
Sn1—O2	2.115 (8)	C5—C8	1.51 (2)
Sn1—O3	2.699 (9)	C5—C6	1.54 (2)
Sn1—Cl1^i^	2.848 (3)	C5—H5	0.9800
Sn2—O1^i^	2.010 (5)	C6—C7	1.31 (2)
Sn2—C12	2.088 (11)	C6—H6	0.9300
Sn2—C11	2.096 (10)	C7—H7	0.9300
Sn2—O1	2.122 (6)	C8—H8A	0.9700
Sn2—Cl1	2.649 (3)	C8—H8B	0.9700
Sn2—Sn2^i^	3.2898 (12)	C9—H9A	0.9600
Cl1—Sn1^i^	2.848 (3)	C9—H9B	0.9600
O1—Sn2^i^	2.010 (5)	C9—H9C	0.9600
O2—C1	1.262 (16)	C10—H10A	0.9600
O3—C1	1.300 (18)	C10—H10B	0.9600
C1—C3	1.55 (2)	C10—H10C	0.9600
C2—C8	1.494 (19)	C11—H11A	0.9600
C2—C7	1.50 (2)	C11—H11B	0.9600
C2—C3	1.51 (2)	C11—H11C	0.9600
C2—H2	0.9800	C12—H12A	0.9600
C3—C4	1.50 (2)	C12—H12B	0.9600
C3—H3	0.9800	C12—H12C	0.9600
			
O1—Sn1—C10	104.6 (4)	C1—C3—H3	108.7
O1—Sn1—C9	105.3 (4)	C5—C4—C3	108.8 (15)
C10—Sn1—C9	145.7 (5)	C5—C4—H4A	109.9
O1—Sn1—O2	81.2 (3)	C3—C4—H4A	109.9
C10—Sn1—O2	100.6 (4)	C5—C4—H4B	109.9
C9—Sn1—O2	100.3 (5)	C3—C4—H4B	109.9
O1—Sn1—O3	131.8 (3)	H4A—C4—H4B	108.3
C10—Sn1—O3	85.5 (4)	C4—C5—C8	98.8 (15)
C9—Sn1—O3	87.1 (4)	C4—C5—C6	88.3 (13)
O2—Sn1—O3	50.6 (3)	C8—C5—C6	97.7 (14)
O1—Sn1—Cl1^i^	74.13 (16)	C4—C5—H5	121.6
C10—Sn1—Cl1^i^	86.0 (3)	C8—C5—H5	121.6
C9—Sn1—Cl1^i^	86.2 (4)	C6—C5—H5	121.6
O2—Sn1—Cl1^i^	155.3 (3)	C7—C6—C5	107.2 (18)
O3—Sn1—Cl1^i^	154.0 (3)	C7—C6—H6	126.4
O1^i^—Sn2—C12	114.6 (4)	C5—C6—H6	126.4
O1^i^—Sn2—C11	111.7 (4)	C6—C7—C2	109.9 (18)
C12—Sn2—C11	133.2 (5)	C6—C7—H7	125.0
O1^i^—Sn2—O1	74.5 (3)	C2—C7—H7	125.0
C12—Sn2—O1	99.8 (4)	C2—C8—C5	102.3 (14)
C11—Sn2—O1	98.5 (4)	C2—C8—H8A	111.3
O1^i^—Sn2—Cl1	79.31 (17)	C5—C8—H8A	111.3
C12—Sn2—Cl1	91.0 (4)	C2—C8—H8B	111.3
C11—Sn2—Cl1	91.0 (3)	C5—C8—H8B	111.3
O1—Sn2—Cl1	153.80 (17)	H8A—C8—H8B	109.2
O1^i^—Sn2—Sn2^i^	38.43 (16)	Sn1—C9—H9A	109.5
C12—Sn2—Sn2^i^	111.3 (4)	Sn1—C9—H9B	109.5
C11—Sn2—Sn2^i^	108.8 (3)	H9A—C9—H9B	109.5
O1—Sn2—Sn2^i^	36.06 (15)	Sn1—C9—H9C	109.5
Cl1—Sn2—Sn2^i^	117.74 (7)	H9A—C9—H9C	109.5
Sn2—Cl1—Sn1^i^	81.42 (7)	H9B—C9—H9C	109.5
Sn2^i^—O1—Sn1	125.1 (3)	Sn1—C10—H10A	109.5
Sn2^i^—O1—Sn2	105.5 (3)	Sn1—C10—H10B	109.5
Sn1—O1—Sn2	129.3 (3)	H10A—C10—H10B	109.5
C1—O2—Sn1	113.3 (10)	Sn1—C10—H10C	109.5
C1—O3—Sn1	83.5 (8)	H10A—C10—H10C	109.5
O2—C1—O3	112.1 (13)	H10B—C10—H10C	109.5
O2—C1—C3	117.7 (16)	Sn2—C11—H11A	109.5
O3—C1—C3	130.1 (15)	Sn2—C11—H11B	109.5
C8—C2—C7	92.7 (16)	H11A—C11—H11B	109.5
C8—C2—C3	102.9 (15)	Sn2—C11—H11C	109.5
C7—C2—C3	96.9 (16)	H11A—C11—H11C	109.5
C8—C2—H2	119.7	H11B—C11—H11C	109.5
C7—C2—H2	119.7	Sn2—C12—H12A	109.5
C3—C2—H2	119.7	Sn2—C12—H12B	109.5
C4—C3—C2	103.5 (14)	H12A—C12—H12B	109.5
C4—C3—C1	118.3 (17)	Sn2—C12—H12C	109.5
C2—C3—C1	108.7 (15)	H12A—C12—H12C	109.5
C4—C3—H3	108.7	H12B—C12—H12C	109.5
C2—C3—H3	108.7		

Symmetry codes: (i) −*x*+1, −*y*+1, −*z*+2.
